# Role of DNA Methyl-CpG-Binding Protein MeCP2 in Rett Syndrome Pathobiology and Mechanism of Disease

**DOI:** 10.3390/biom11010075

**Published:** 2021-01-08

**Authors:** Shervin Pejhan, Mojgan Rastegar

**Affiliations:** Regenerative Medicine Program, and Department of Biochemistry and Medical Genetics, Rady Faculty of Health Sciences, Max Rady College of Medicine, University of Manitoba, Winnipeg, MB R3E 0J9, Canada; pejhans3@myumanitoba.ca

**Keywords:** epigenetics, DNA methylation, MeCP2 isoforms, MeCP2E1, MeCP2E2, BDNF, *miR132*, brain development, Rett syndrome, RTT pathobiology

## Abstract

Rett Syndrome (RTT) is a severe, rare, and progressive developmental disorder with patients displaying neurological regression and autism spectrum features. The affected individuals are primarily young females, and more than 95% of patients carry *de novo* mutation(s) in the Methyl-CpG-Binding Protein 2 (*MECP2)* gene. While the majority of RTT patients have *MECP2* mutations (classical RTT), a small fraction of the patients (atypical RTT) may carry genetic mutations in other genes such as the cyclin-dependent kinase-like 5 (*CDKL5)* and *FOXG1*. Due to the neurological basis of RTT symptoms, MeCP2 function was originally studied in nerve cells (neurons). However, later research highlighted its importance in other cell types of the brain including glia. In this regard, scientists benefitted from modeling the disease using many different cellular systems and transgenic mice with loss- or gain-of-function mutations. Additionally, limited research in human postmortem brain tissues provided invaluable findings in RTT pathobiology and disease mechanism. MeCP2 expression in the brain is tightly regulated, and its altered expression leads to abnormal brain function, implicating MeCP2 in some cases of autism spectrum disorders. In certain disease conditions, MeCP2 homeostasis control is impaired, the regulation of which in rodents involves a regulatory microRNA (*miR132*) and brain-derived neurotrophic factor (BDNF). Here, we will provide an overview of recent advances in understanding the underlying mechanism of disease in RTT and the associated genetic mutations in the *MECP2* gene along with the pathobiology of the disease, the role of the two most studied protein variants (MeCP2E1 and MeCP2E2 isoforms), and the regulatory mechanisms that control MeCP2 homeostasis network in the brain, including BDNF and *miR132*.

## 1. Introduction to Rett Syndrome

Rett Syndrome (RTT) is a neurological disease in females that is commonly diagnosed in female infants by 1–2 years of age. The disease mainly affects brain development and the symptoms progress as the patient grows up. In most cases, children seem to be normal at the time of birth and up to six months of age, after which they start exhibiting specific symptoms of the disease. RTT patients display a wide range of neurological and developmental impairments that require continuous care throughout their life. While RTT is commonly considered a monogenic disorder caused by methyl-CpG-binding protein 2 (*MECP2*) gene mutations, in a small percentage of cases, the disease is associated with mutations in some other genes. In this review, we will mainly focus on Rett Syndrome caused by *MECP2* mutations. This includes the history of the disease, clinical characteristics of RTT patients, MeCP2 as an epigenetic factor, MeCP2 mutations and homeostasis regulation, MeCP2 targets focusing on BDNF-*miR132* and relevant signaling pathways, as well as disease pathobiology.

## 2. History of Rett Syndrome

Over half a century ago in 1954, Dr. Andreas Rett who was a pediatrician from Austria noticed similar winding hand motions in two young girls waiting for a visit in his clinic. The clinical and developmental histories of these two patients were also similar. With further investigations, Dr. Rett found 6 other girls with the same disorder in his own practice and 22 patients during his travels throughout Europe. Twelve years after that eye-catching coincidence in the waiting room of his clinic, Dr. Rett reported the clinical entity in the German medical literature [1]. Seventeen years later, Dr. Bengt Hagberg, a neurologist from Sweden, in collaboration with his colleagues, attributed Rett’s name to this Syndrome, mainly overlooked because of the language of the first report. The medical community recognized Rett Syndrome through an English report of 35 RTT cases that were reported by Dr. Hagberg in 1983 [2]. In 1992, the *MECP2* gene was first reported by Dr. Adrian Bird and his team at the University of Edinburg, UK [3]. Seven years later, *MECP2* mutation was discovered to be the underlying cause of RTT pathophysiology by Dr. Huda Zoghbi and colleagues. They showed that mutations in the *MECP2* gene are causative for the majority (over 90%) of RTT cases [4]. Soon after finding the genetic basis of RTT, in 2001, the first animal model of RTT became available [5]. Since then, several groups have worked to elucidate the pathophysiology of the disease and have run trials for therapeutic purposes [6].

## 3. Clinical Features, Diagnosis, and Histopathology of RTT

Rett syndrome is a neurodevelopmental disorder that progresses with age and is mainly seen in females (1:10,000 live female-births) [7]. Classical RTT is caused by *MECP2* mutations with relatively well-defined characteristics, while atypical RTT is characterized by early onset of seizers and developmental delay. Atypical RTT can be seen in patients with genetic mutations in other genes, such as cyclin-dependent kinase-like 5 (*CDKL5*) and FOXG1 [8,9]. In classical RTT, neurodevelopmental progression seems to be normal during the first six to eighteen months, although subtle symptoms such as muscle hypotonia and deceleration of head growth are usually present earlier in their life but frequently ignored. Delay, stagnation, or regressions in motor development are among the most frequent complaints that bring patients to medical attention. General growth delay, weight loss, and a weak posture caused by muscle hypotonia are other common findings at this stage [10,11].

As RTT progresses, stereotypic hand wringing or washing movements replace purposeful use of hands. Abnormal gait with lack of coordinated movements of the upper extremities in addition to social withdrawal and loss of verbal communications are other common symptoms in RTT patients. The autistic features such as poor response to environmental stimulations become less prominent and are replaced by signs of mental retardation as the child grows up [7]. RTT patients also suffer from autonomic perturbations including breathing abnormalities (e.g., breath holding, periods of hyperventilation, and apnea) [12], cardiac arrhythmias (prolonged QT syndrome) [13], and gastrointestinal dysfunction [14].

Seizures ranging from easily controlled to intractable epilepsy are common in RTT and could be seen in more than 80% of cases [15]. Age of onset of seizures depends on the type of *MECP2* gene mutation, and its severity tends to decrease after the teenage years and into adulthood [15]. Ataxia (gross lack of coordination in muscle movements) and gait apraxia (inability to perform learned movements) accompany mental detriment. Devastating motor dysfunction makes RTT patients wheelchair-bound during the teenage years and as they enter adulthood. Parkinson-like features could be added to the clinical manifestations. Skeletal deformities such as scoliosis and osteopenia in RTT patients are partly caused by locomotor difficulties and sedentary state. Decreased muscle tone can also be responsible in this process [14]. Despite poor physical condition, RTT patients typically survive into adulthood (70% up to 45 years) and even up to 70 years of age. Cardiorespiratory compromise is the leading cause of death in RTT [16]. The three main atypical forms of RTT are well-maintained speech irregularity, seizure with an early onset, and the congenital variant [7,17].

### Gross and Microscopic Features of RTT

Microcephaly is the main finding in gross pathology of Rett Syndrome. RTT patients show a normal head circumference at birth but begin to display deceleration after 2-3 months. The reduction in brain weight is not universal, and cerebral hemispheres are relatively smaller compared to the cerebellum and compared to non-RTT conditions [18]. Prefrontal, posterior frontal, and anterior temporal brain regions show smaller volumes in neuroimaging, while posterior temporal and posterior occipital regions are relatively preserved [19,20]. In general, microscopic evaluations of RTT brains have not recognized degeneration, demyelination, or gross malformative processes. A reduction in gross brain volume is associated with small and compacted neurons that also suffer from decreased dendritic complexity, reduced neurites, and a lower level of synaptic density in the cerebral cortex [21]. Reduced melanin and tyrosine hydroxylase staining in the midbrain and substantia nigra, and altered contour and appendages in neurons of the globus pallidus are examples of findings in other regions of the brain in RTT Syndrome [22,23]. Vagal tone abnormalities that have been found in functional studies of the vagus nerve are in line with the autonomic impairments in RTT. Aberrations in serotonin receptors as well as substance P content have also been reported in brain stem studies [18] Substance P is a neuropeptide acting as a neurotransmitter and neuromodulator, best reported for its role in the transmission of pain stimuli in the peripheral nervous system, but it participates in behavioral responses as well as neuronal survival in the central nervous system [24]. The altered sensitivity of Rett Syndrome patients to pain can be related to the abnormality in substance P. There are also studies that suggest a role for MeCP2 in pain perception [25,26,27].

## 4. Genetic Basis of Rett Syndrome: Link between Genotype and Phenotype

RTT is an X-linked disease and predominantly affects females; however, it can also be seen in three groups of male patients. Patients in the first group have a mutation that causes classical RTT in females, though these boys have severe neonatal encephalopathy and a lifespan of less than one year and commonly die early during childhood. The diluting effect of X chromosome aneuploidy (Klinefelter syndrome; 47 XXY)) or somatic mosaicism may be associated with a milder phenotype, similar to atypical RTT [28,29]. The second group presents mutations which are different from typical/classical mutations in female patients but are more compatible with life into adulthood. The last group shows mutations in genes other than *MECP2* such as *CDKL5* or *FOXG1* and are associated with RTT-like symptoms in males [30].

**Figure 1:**
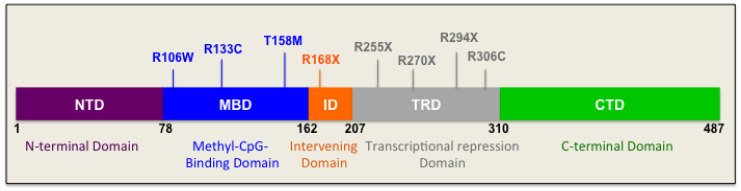
Schematic representation of the MeCP2 protein domains: A simplified representation of MeCP2 protein domains is shown. The drawing is not in scale. Main point mutations that are among the most common mutations are indicated. The name of each domain is abbreviated inside the image, and the full name is provided underneath the respective domain.

*De novo* mutations in the *MECP2* gene positioned in the X-chromosome (Xq28) are the source of 95% of cases of typical RTT and over half of atypical RTT events [31]. In general, the majority of such *de novo* mutations occur in the germline of the father [32,33], which partially explains the female predominance of RTT. In rare familial cases of Rett Syndrome, maternal skewing of X-chromosome inactivation (XCI) could pass the same *MECP2* gene mutation from an asymptomatic female carrier to her children [34]. Having one copy of X-chromosome, most male patients present severe symptoms including infantile encephalopathy and death during early childhood [34]. From more than 4600 identified variants in the *MECP2* gene, over 70% have an association with RTT. However, the causative role of *MECP2* mutation has not been shown for all of them. Eight of these mutations account for about 47% of all mutations that include three mutations in the methyl-CpG-binding domain (R106W, R133C, and T158M), one mutation in the intervening domain (ID) (R168X), and four mutations in the transcriptional repression domain (R255X, R270X, R306C, and R294X) (Figure 1). Some of the identified RTT-associated *MECP2* mutations impact MeCP2 protein stability, which may be partly involved in disease biology [35,36,37]. As indicated, mutations in two other genes, *FOXG1* and *CDKL5*, can cause atypical forms of RTT [38].

RTT patients exhibit a high range of symptomatic variation, from mild to very severe symptoms. While dividing RTT into classical and atypical forms has decreased the differences within each category, there is still phenotypic variation in each group and even in one patient over time. X-chromosome inactivation is suggested as one of the main contributors of phenotypic variability [39]. While most classical RTT patients show balanced XCI pattern in the brain [40], non-random XCI has also been reported [41]. Somatic mosaicism is another potential source of phenotypic variability [42]. Finally, specific types of *MECP2* mutations can also affect protein functions differently and can lead to different severities of phenotypes. For instance, severe RTT caused by T158M mutation (affecting the methyl-CpG-binding domain of MeCP2) may be due to interrupted MeCP2 binding to methylated DNA, resulting in changes in the transcriptional control of its target gene [43,44], whereas C-terminal deletions may be associated with milder phenotypes [45].

### 4.1. Mutations in the N-Terminal Domain

From the hundreds of *MECP2* mutations found in RTT patients, less than 1% target exon 1, which means MeCP2E1 dysfunction is sufficient to cause Rett Syndrome [46,47,48]. While MeCP2E2-specific mutations have not been described in RTT, it has been shown that the expression of MeCP2E2 can improve some of the RTT phenotypes in *Mecp2*-null mice. Isoform-specific MeCP2 transgenic lines were used in this study to rescue neurophenotypes exhibited by *Mecp2*-null mice (*Mecp2*^−/Y^) [49]. Mutations affecting parts of the N-terminal domain (NTD) region, which are shared by both MeCP2 isoforms, have been described in RTT patients [38].

### 4.2. Mutations in the Methyl-CpG-Binding Domain

Thr158, Arg133, and Pro152 within the MeCP2 methyl-CpG-binding domain (MBD) region are three common spots for RTT-related mutations. T158M located in the MBD region of the protein is the most frequent mutation that can cause RTT (approximately 9%). R133C and R106W from the MBD region are ranked 7th and 10th in the list of RTT-causing mutations [38].

### 4.3. Mutations in the Intervening Domain

The intervening domain is the region that links MBD and Transcription Repression Domain (TRD) and is the location for R168X, a truncating mutation that is the second most common mutation in RTT patients [38]. A few mutations in the ID region of MeCP2 such as R190H and R190C have been also associated with Schizophrenia [50,51]. One of the RTT brain samples in our recent studies was shown A201V mutation. This is the 21st most common *MECP2* mutation found in 0.61% of RTT patients. However, it is unclear if it has a cause effect in this syndrome or is just a polymorphism found in association with RTT [38].

### 4.4. Mutations in the Transcriptional Repression Domain

TRD binds with partner proteins such as co-repressor Sin3a, histone deacetylases (HDACs) [52], and NCoR/SMRT co-repressor [53]. R255X, R270X, and R294X, the three most common truncating mutations after R168X (from ID), are located within TRD. Moreover, R306C, which is the second most common missense mutation after T158M (from MBD), is located in TRD [38]. Because of the R306C mutation, MeCP2 loses its interaction with NCoR/SMRT [53].

### 4.5. Mutations in the C-Terminal Domain

The MeCP2 C-terminal domain (CTD) might play a role in binding to histones [54]. This domain also contains S421, a serine residue that can become phosphorylated during neural activity [55], resulting in transcriptional activation of the Brain-derived neurotrophic factor (*Bdnf*) [56]. L386fs, the most common frameshift mutation in RTT patients, is located in CTD. E397K, which is the sixth most common RTT-associated substitution, is also located in CTD.

## 5. Biological Systems to Study Rett Syndrome

RTT has a monogenic cause resulting from mutations in the single *MECP2* gene [4]. This has motivated the generation of many RTT transgenic animals [5,57,58] and cellular [59,60,61] model systems for the disease. Based on their genetic modifications, the models can be categorized as (1) *Mecp2*-deficient models such as *Mecp2* constitutive knockout mice [5,58] or brain region/cell type-specific deletion of *Mecp2* [62,63], and (2) *Mecp2* mutant models such as knock-in mouse models with specific *Mecp2* mutations [64,65]. These animal models show different phenotypes and have different lifespans [66]. While very useful, one may need to be aware of some potential caveats in the interpretation of RTT animal model studies. First, while *Mecp2*-heterozygous female mice are more directly representative of RTT condition, male *Mecp2*-null mice are easier to work with and are more frequently used. Second, compared to humans, mice show noticeable symptoms for MeCP2 loss-of-function later in the course of development [67].

With regards to in vitro human RTT cellular model systems, MeCP2-deficient cultured neurons derived from either human-induced pluripotent stem (iPS) cells or embryonic stem cells have been used [59,60]. Murine cellular model systems include differentiated cells from embryonic stem cells or primary neural stem cells [67]. For detailed information on different RTT model systems, we refer the readers to excellent recent reviews [68,69,70,71,72,73,74,75,76,77,78,79].

## 6. Epigenetic Regulation Mechanisms and Role in Controlling MeCP2 Homeostasis Network

MeCP2 is an epigenetic factor and is among the most-studied proteins that are involved in epigenetic control. Epigenetic mechanisms regulate gene expression without direct change in the underlying DNA sequences. Such mechanisms include chromatin remodeling, DNA methylation, RNA modifications, histone post-translational modifications (PTM), and the activity of different types of regulatory RNAs [80]. It has been shown that epigenetic mechanisms play major roles in development, aging, and disease conditions [80,81]. Below, we provide a brief description of the most-studied mechanisms of epigenetic regulation, epigenetic control of MeCP2, and its homeostasis network.

### 6.1. Chromatin Remodelling

In eukaryotic cells, the genomic material is composed of DNA and DNA-bound proteins (called histones), together making up the “chromatin” structure. A 147-bp stretch of DNA folded around a histone octamer (consisting of 2 H2A-H2B dimers and 2 copies of each histone H3 and H4) and the histone octamer, forming the basic and repetitive unit of the chromatin structure, known as a “nucleosome”. A stretch of 20-to-50 bp linker DNA associates the nucleosomes together. This linker DNA is accessible to DNA binding proteins, but nucleosomes are considered negative regulators of gene transcription. It has been shown that a group of proteins can modulate gene expression through repositioning of nucleosomes and remodeling of the chromatin in the promoter of specific genes [82]. This process has been shown to be important during neurodevelopment [83,84,85].

### 6.2. Histone Post-Translational Modifications

The N-terminal tail of histones mainly includes amino acids that are subjected to various PTMs. Specific amino acids such as lysine are commonly the target for acetylation, phosphorylation, methylation, sumoylation, and ubiquitination, whereas arginine can be methylated or ADP-ribosylated [86,87]. These histone marks can affect transcriptional activity of the genes by recruiting co-activator or co-repressor complexes [88]. Various histone PTMs play key roles in important processes such as demarcating euchromatin and heterochromatin regions. For example, facultative heterochromatin, which contains selectively silenced genes, is enriched for H3K27me3, whereas the constitutive heterochromatin with permanently repressed genes (like centromere) contains abundant H3K9me3 [89,90,91,92].

### 6.3. Noncoding RNAs

In the human genome there are approximately 21,000 protein-coding genes, which is similar to less complex species; however, there are tens of thousands of noncoding RNAs (ncRNAs) that play regulatory roles in physiological complexity of humans and other mammals [93]. Small RNAs (approximately 20–30 nucleotides in length) include small interfering RNA (siRNA), microRNAs (miRNAs), and Piwi-interacting RNAs (piRNAs) that modulate gene expression in a way that is specific to their target sequences. Long ncRNAs (typically >200nt) are among the important players of transcriptional regulation at multiple levels [85,94,95].

### 6.4. DNA Methylation

DNA methylation is perhaps one of the most important types of epigenetic modifications primarily characterized as the attachment of a methyl group at the 5th carbon of a cytosine, known as 5-methyl cytosine (5-mC). This methylation is commonly in the context of cytosine guanine dinucleotide (CpG). DNA methylation can also happen in the non-CpG context, targeting other nucleotides (adenine, guanine, and thymine) [91]. While DNA methylation first was recognized as a marker for gene inactivation, later, it became clear that, in the context of 5-hydroxy methyl cytosine (5-hmC), it could activate gene expression [91,96]. Epigenetic modifications are mediated by specific enzymes known as writers, recognized by effector proteins known as readers, and the reversible marks can be removed by another set of enzymes called erasers [80].

### 6.5. Writers of DNA Methylation

The process of DNA methylation is facilitated by DNA methyl transferase (DNMT) enzymes that include DNMT3A and DNMT3B. These enzymes are in charge of *de novo* DNA methylation, while DNMT1 is the maintenance DNMT. DNMT enzymes are important for proper development, and their impairments are reported in different diseases. For instance, DNMT1 mutation is associated with “hereditary sensory neuropathy with hearing loss and dementia type IE” [97,98].

### 6.6. Erasers of DNA Methylation

DNA demethylation can occur in a passive way when methylation marks dilute and fade from one cell division to the next. This happens in early stages of development in which production of DNMTs has not yet started and the DNMT1 that originated from oocytes is diluted by cell division. There is also an active DNA demethylation catalyzed by the activity of the Ten-eleven translocation (TET) family of proteins that transform a 5-methylcytosine into a 5-hydroxymethylcytosine, which could undergo multiple steps to finally become an unmethylated cytosine [80,91,97]. DNA methylation is read and interpreted by different families of proteins that recognize this epigenetic modification and bind to it. The Methyl-CpG-binding protein (MBP) family include multiple members, with MeCP2 being the prototype member of this group of proteins [81,99].

### 6.7. Methyl-CpG-Binding Protein Family

This family of DNA methylation readers is characterized by a methyl-CpG-binding domain that facilitates protein binding to methylated DNA. From its 11 members, MeCP2, MBD2, and MBD3 are preferably associated with methylation of the promoters and generally suppress gene transcription. MBD1 mostly functions through histone modification and heterochromatin formation, and MBD4 takes part in DNA repair. Dysregulation or mutations of MBD proteins are present in a variety of cancers as well as neurologic disorders such as RTT [81,100,101]. MeCP2 is the best-studied member of a family of proteins that bind to methylated CpG DNA templates without sequence specificity. While the first member of this group (MeCP1) needs at least 12 symmetrical methylated CpG, the second and most abundant protein of this group (MeCP2) is able to bind a single methylated CpG pair [102,103].

### 6.8. MECP2/Mecp2 Gene Structure and MeCP2 Protein

In humans, the *MECP2* gene is located on the long arm of the X-chromosome (Xq28), while in mice, it is positioned at the XqA7.3. In mice (*Mecp2*) and humans (*MECP2*), the gene consists of 4 coding exons and 3 introns. Three polyadenylation sites in its 3′UTR result in mRNA transcripts with varying lengths. The two translational start sites at exon one and two give rise to the common splice variants of the protein that differ only at their N-terminal domains. The MeCP2E1 isoform results from the coding sequences of exons 1, 3, and 4, and its transcripts are reported to be the main isoform in the brain. MeCP2E2 is encoded by exons 2, 3, and 4, and its transcript level has been reported to be higher than *MECP2E1* in the liver, placenta, prostate gland, and skeletal muscles [46].

MeCP2 protein is composed of 5 major functional domains including an N-terminal domain, a methyl-CpG-binding domain, an intervening domain, a transcription repression domain, and finally a C-terminal domain, as shown in Figure 1. Three AT hook domains, which exist within ID, TRD, and CTD, make binding to AT-rich DNA possible [104]. In general, MeCP2 is known to as a nonstructured and disordered protein due to its major unstructured format (approximately 60%) [105]. From the two MeCP2 isoforms, MeCP2E1 (previously called MeCP2B or MeCP2α) has 21 distinctive residues at its N-terminal region and an acidic isoelectric point (pI) of 4.24. The other isoform that was discovered first, MeCP2E2 (previously called MeCP2A or MeCP2β), has 9 exclusive residues at the N-terminal region and a basic pI of 9.5. The two MeCP2 isoforms show differential chromatin binding activities [106].

Dr. Bird and his team suggested that the presence of an upstream open reading frame in *MECP2E2* could have an inhibitory effect on protein translation and could result in more abundant MeCP2E1 [107]. However, due to the unavailability of specific antibodies and reagents that recognize endogenous MeCP2E1 and MeCP2E2 isoforms, research was limited to their transcript analysis until nine years ago when our team reported the first generation and validation of the MeCP2E1-specific isoform antibody [108], and subsequently, we reported both E1- and E2-specific antibodies [109]. Using these newly developed tools at the time, our lab reported that MeCP2E1 has a relatively uniform distribution across different brain regions such as the cortex, hippocampus, thalamus, brain stem, and cerebellum, while MeCP2E2 is differentially enriched in various brain regions of a mouse [109]. Our results revealed that MeCP2E1 has an earlier onset of expression in the brain during development and that MeCP2E2 is expressed later during brain development, peaking postnatally with a brain region-specific pattern of detection. Recently, we analyzed this least-studied difference between MeCP2E1 and MeCP2E2 isoforms in the human brains [110].

### 6.9. MeCP2 Expression and Regulation

Even though the brain-specific role of MeCP2 has been broadly studied in the context of neurological characteristics of RTT, MeCP2 has been found in different organs from the lung and spleen with high expression levels to the liver, heart, and small intestine with lower expression levels [40]. In the brain, MeCP2 is detected in neurons, neural stem cells, glia including astrocytes and oligodendrocytes [43,111,112,113], and microglia [114]. Selective MeCP2 deficiency in these cell types has caused neuronal abnormalities, which could be then resolved by re-expression of MeCP2 in these cells [115,116]. In general, DNA methylation is an important mechanism by which MeCP2 isoforms are regulated in murine neural stem cells, neurons, and astrocytes as well as in different regions of the brain in adult mice in a cell type- and sex-dependent manner [96,109,113,117,118,119].

**Figure 2:**
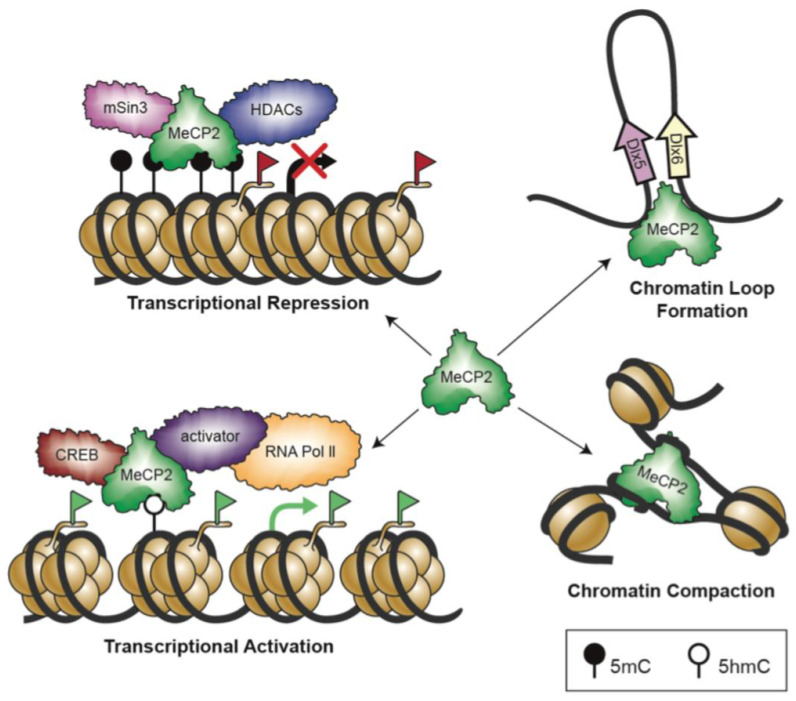
Schematic representation of different MeCP2 functions in brain cells: this simple hypothetical cartoon illustrates some of the conceptual functional properties of MeCP2, through which it controls gene regulation. Figure is adapted and modified from Zachariah and Rastegar [127]. Red and green flags refer to inactive and active histone marks, respectively.

In terms of its function, MeCP2 was originally considered an inhibitor of gene regulation through interaction with a co-repressor complex composed of mSin3A, a transcriptional repressor, and HDACs. Such regulatory role can lead to compaction of the chromatin structure and gene silencing [120,121]. NCoR/SMRT is another, more recently found co-repressor complex that has a specific binding domain in the TRD region of MeCP2 [53]. In contrast to primary findings, researchers have shown that MeCP2 can also be a transcriptional activator by recruiting cAMP response element-binding protein (CREB) [122]. It has been also suggested that MeCP2 can play the role of a transcription activator when it binds to 5-hydroxymethylcytosine, which is a common modification of DNA in the brain and is enriched in active genes [123]. Other studies suggest that MeCP2 can play the role of a global regulator of chromatin. The MeCP2 level in neurons is almost similar to that of histones. In addition, it can bind to non-methylated DNA [124] and compact nucleosomes in a manner similar to histone H1 [125]. MeCP2 may also affect its targets such as *DLX5* by making a loop in DNA [126] (Figure 2).

MeCP2 is regulated transcriptionally and post-transcriptionally by multiple mechanisms. Positive and negative regulatory factors upstream of the *MECP2* promoter region can regulate its expression. There are also silencers and enhancers in the region that can act as *cis*-regulatory elements for the *MECP2* gene [113,128]. In addition, there are polyadenylation sites at 3′UTR of the *MECP2*/*Mecp2* gene which are responsible for different lengths of transcripts in a tissue-specific manner. *Trans*-acting factors involved in polyadenylation can bind to these sites [129,130]. Similar to other genes, epigenetic factors such as microRNAs and histone PTMs can also affect MeCP2 regulation [81]. Our lab has already shown how DNA methylation can affect the expression of *Mecp2* isoforms during in vitro neural stem cell differentiation [113]. Furthermore, we have reported how an environmental insult such as ethanol exposure can cause misexpression of *Mecp2*/MeCP2 in differentiating brain cells through deregulation of two types of DNA methylation (5-mC and 5-hmC) [96]. Recently, we reported that not only MeCP2 but also other DNA methylation-related factors show strain- and sex-specific regulation in mice [117,118,119].

## 7. MeCP2 Target Genes: A Focus on BDNF and Its Related Signaling Cascades

Studies on MeCP2 target genes show little overlap, and association of these target genes with RTT has not been established in most cases [127]. MeCP2 is capable of having either an activator or a repressor effect on these studied targets not only as a transcriptional regulator but also as an epigenetic modulator that can affect RNA splicing and chromatin structure as explained earlier [81]. Genes such as *Dlx5*, *Fgf2–5*, *Fut8*, and *Nf1* have shown alterations in splicing observed in an RTT mouse-model (*Mecp2^308/y^*) [131]. Brain-derived neurotrophic factor is an important and perhaps the most studied target of MeCP2. Its cross-talk with MeCP2 has been studied mainly in animal models [56,132]. MicroRNAs (miRNAs) are other important targets for MeCP2 and its regulations. Several miRNAs have shown altered expressions in RTT mouse models [96,133].

### 7.1. Brain-Derived Neurotrophic Factor

Brain-derived neurotrophic factor is a well-known member of the neurotrophin family of growth factors. Neurotrophins together with tropomyosin receptor kinases (TRKs) and low-affinity nerve growth factor receptor (p75) (also called p75 neurotrophin receptor (p75NTR) regulate survival, maturation, and differentiation of neurons and participate in synaptic development and neural plasticity [134,135,136]. In general, neurotrophin genes have multiple 5′ exons with their main regulatory elements (promoters) that would recruit RNA polymerase machinery to initiate transcription of distinct mRNAs. A 3′ exon that is common among all transcripts includes an open reading frame encoding the precursor peptide pre-pro neurotrophin. Despite similarities among the gene structures of different neurotrophin family members, the *BDNF*/*Bdnf* gene has one of the most complex structures, which is closely conserved between humans and rodents. Mice, rats, and humans have at least eight homologous exons in common, which are regulated by alternative upstream promoters. The multi-promoter character of *BDNF*/*Bdnf* is suggestive of the additional flexibility of BDNF expression in response to a diverse range of stimuli [137].

The human *BDNF* gene spans about 70 kb on chromosome 11 within the region p13-14 and includes 11 exons (known as I–IX, plus Vh and VIIIh). Some exons have different subsets that are labeled a, b, c, and d. The protein coding sequence is located within exon IX, and different upstream promoters of alternatively spliced exons yield several *BDNF/Bdnf* transcripts. There are two polyadenylation sites for exon IX that double the number of transcripts by generating short and long splicing variants. Despite the existence of different transcripts, all the mRNAs encode for a single protein. The apparently redundant generation of transcripts controls the requirements of context- and cell type-specific demands [138]. For example, while exons I, IV, V, VII, and IX in a mouse are activated by DNA methylation, a different group of exons (III, VIII, and IX) are induced by inhibitors of histone deacetylase [139]. In addition, it has been shown that transcript variants with short 3′UTR stay in the soma and regulate neuronal survival, while transcripts containing the long 3′UTR are preferentially localized in the dendrites to modulate synaptic plasticity [140,141,142].

#### 7.1.1. BDNF Signaling

BDNF binds the tropomyosin-related kinase B (TrkB) with specific/high affinity. This leads to TrkB dimerization and auto-phosphorylation, activating several downstream signal transduction cascades. That includes the mitogen-activated protein kinase (MAPK) which promotes neural differentiation, the phosphatidylinositol 3-kinase (PI3K) that promotes growth and survival of neurons, and the phospholipase Cγ (PLCγ) pathway that promotes synaptic plasticity [143]. Similar to other neurotrophins, BDNF can also bind to p75 (also known as p75NTR). p75NTR is part of the tumor necrosis proteins that can activate several signaling pathways including programmed cell death, when p75NTR is activated in the absence of TrkB signaling [144].

The cooperation of p75NTR and TrkB receptors increases the affinity of mBDNF for the complex, and the pro-survival, growth-related signaling will be enhanced [145,146]. On the contrary, when p75NTR forms a heterodimer with sortilin (a transmembrane protein that regulates neurotrophin sorting), the resulted complex acquires a higher affinity for Pro-BDNF. Cell-death signaling will then be activated through the induction of several proapoptotic pathways [147]. Therefore, the biological role of the pro-peptide is beyond the traditional assistance in folding of the mature protein. It has been shown that the BDNF pro-peptide is biologically active as a synaptic modulator that facilitates long-term depression when directly binding the p75NTR receptor [148]. From different downstream signaling cascades, the PLCγ pathway has been suggested to mediate rapid BDNF-related effects that happen within seconds to minutes, while the other two (MAPK and PI3K) work more slowly through changes in gene transcription [145].

When TrkB becomes phosphorylated at Tyr^490^ and Tyr^515^, its affinity for Src homology 2 domain containing adaptor protein (Shc) increases. Upon binding to the specific phosphorylated sites, the growth factor receptor bound protein 2 (Grb2) is recruited. Grb2 makes a complex with Son of Sevenless (SOS), which is an exchange factor for the RAS protein. RAS acts upstream of ERK (extra cellular signal-regulated kinase1/2) in the MAK cascade, activating RAF protein Ser/Thr kinase. That in turn leads to the activation of MEK (MAP kinase/ERK kinase), which can activate ERK1/2. Active ERK can then transfer to the nucleus, activating transcription factors such as CREB. The phosphorylated CREB can bind to the *Bdnf*/*BDNF* promoter, inducing its transcription. The BDNF-ERK-CREB signaling pathway plays a major role in cell survival, synaptic structure, and plasticity [149,150,151,152]. TrkB phosphorylation at Tyr^816^ generates inositol 1, 4, 5-triphospahte (IP3) and diacylglycerol (DAG). IP3 is responsible for Ca^2+^ release from internal sources, leading to the activation of Ca^2+^/calmodulin-dependent protein kinases. This results in CREB phosphorylation, and a cascade is initiated that continues similar to the MAPK pathway [153,154]. Activation of PI3K by BDNF can be associated with the combined action of RAS [155] that can also activate AKT, an important kinase that phosphorylates mTOR (mammalian Target of Rapamycin). The signal transduction cascades that activate the mTOR pathway are involved in the regulation of protein translation, and that is where BDNF may become involved in local protein synthesis [156,157]. Of note, studies from our team showed an impaired mTOR pathway in the human RTT brain with compromised multi-step regulation of the processes that control ribosome biogenesis. Such abnormalities were found in the cerebellum of RTT patients with common (T158M and R255X) and uncommon (G451T) mutations. A regulator of ribosomal RNA processing (Nucleolin) was found to be abnormally redistributed in the neurons of the cerebellum of a T158M patient compared to a control. This was obviously detected in the neurons of the molecular, granular, and Purkinje cell layers of the cerebellum in this patient. To our surprise, null *Mecp2*-deficient male and heterozygote female mice showed no abnormalities in the Nucleolin neuronal distribution in the cerebellum [158]. This study suggested that a lack of the *Mecp2* gene in transgenic mice may not fully recapitulate all molecular deficiencies of the human RTT brain, highlighting the importance of side-by-side analysis of RTT model systems and postmortem brain from RTT patients.

#### 7.1.2. *BDNF/Bdnf* Regulation

Membrane depolarization in neurons could be induced by sensory stimuli [159,160,161], activation of glutamate receptors [162,163,164], or seizure [165], with a positive regulatory role on *BDNF* transcription. It has been shown that binding of cAMP response element-binding protein (CREB) to cAMP/Ca^2+^-response element (CRE) in promoter IV of the human *BDNF* gene is critical for activity-dependent transcription from this promoter. Human promoter IX can be also induced by neuronal activity. CRE and PasRE (basic helix-loop-helix-PAS transcription factor response element) contribute to the induction of this promoter [166]. *BDNF/Bdnf* transcription is reported to be regulated at least in part by epigenetic factors. Decreased methylation of cytosine residues in CpG dinucleotides at *BDNF/Bdnf* promoter IV has been shown in transcription induction of the gene by neuronal activity. That is where MeCP2 can play its role as a transcriptional regulator by binding to methylated DNA in the region of *BDNF/Bdnf* promoter IV [56].

A few studies have examined histone modifications as another epigenetic regulator of *BDNF* transcription. For instance, an increase in histone methyl transferase H3K4 tri-methylation, a marker of active chromatin, has been shown at *BDNF* promoters I and IV during BDNF upregulation in the course of transition from the fetal to childhood and/or young adult stages [167]. MicroRNAs (discussed in next section) are among the epigenetic regulators that can modulate *BDNF* expression. Several microRNAs have been studied in this regard. It has been shown that *miR-1*, *miR-106*, *miR-155*, and *miR191* can suppress *BDNF* gene expression by binding to the *BDNF* 3′UTR [168]. There is a long list of miRNAs predicted to target *BDNF*, and *miR132* has been reported to regulate BDNF [169].

#### 7.1.3. Role of MeCP2 in BDNF Regulation

There is still controversy about BDNF regulation by MeCP2. Earlier studies were more in favor of a repression model. It was reported that membrane depolarization could release MeCP2 from its binding site at *Bdnf* promoter IV, resulting in *Bdnf* transcriptional activation [56,170]. MeCP2 phosphorylation at Ser^421^ [55] and decreased DNA methylation at *Bdnf* promoter IV [132] induced by neuronal depolarization cause MeCP2 dissociation along with its co-repressors (Sin3a and HDAC1) from *Bdnf* promoter IV. Other studies showed reduced *Bdnf* transcript and protein in the *Mecp2*-null mice (hemizygous *Mecp2^tm1.1Jae^* mutant males of the Jaenisch strain and cre93 *Mecp2^−/^*^y^), suggesting an activator role for MeCP2 in *Bdnf* transcription [171,172].

There are other models that suggest a dual role for MeCP2 in BDNF regulation, explaining these discrepancies [173]. Based on a model studied in SH-SY5Y neuroblastoma cells, MeCP2 remains bound to its target genes and the regulatory complexes, which are recruited by its phosphorylation and may activate or repress expression of these target genes [174]. MeCP2 phosphorylation is not the only contributing factor in this dual operating model. Other epigenetic mechanisms can also play a role. For instance, it has been shown that transcription of some of the microRNAs that target the 3′UTR of *Bdnf* transcripts are controlled by MeCP2 [96]. Furthermore, changes in MeCP2 might affect not only the expression of *BDNF*/*Bdnf* at the transcription level but also the translation or stability of the BDNF protein. That might explain some of the discrepancies between the different models that have studied BDNF at the transcript or protein levels [175].

#### 7.1.4. BDNF and Pathophysiology of RTT

Studies on RTT mouse models have shown reductions in BDNF expression after the first 3–4 postnatal weeks at the same time that RTT-like features start to appear. The decrease manifests first in caudal parts of the brain (brainstem and cerebellum), and gradually, the entire brain is involved [173]. There are controversial results about compromised BDNF levels in the brain of RTT patients. While two reports show that the level of BDNF protein in cerebrospinal fluid (CSF) and serum of RTT patients is comparable with unaffected controls [176,177], there are other studies that describe lower transcript levels of BDNF in RTT brain samples compared to controls. Technical limitations especially for the assessment of protein levels might be the reason behind the disparities [178,179]. Recent work from our team shows that BDNF transcripts are significantly reduced in RTT brains while the protein levels remain unchanged, with differential detection pattern at least in the Purkinje cells of the cerebellum [110,180]. In addition to the aforementioned arguments, the functional magnitudes of impaired BDNF function are not fully comprehended. Transgenic approaches to generate mouse models with *Bdnf* loss-of-function have established that *Bdnf*-deficiency leads to comparable phenotypes such as *Mecp2* deficiency, smaller brain and nerve cells (neurons) with neurite arborisation, compromised hippocampal long-term potentiation in *Bdnf*-deficient or Trkb-deficient mice [181], irregular breathing, and impaired locomotion [182,183]. Moreover, *Bdnf* overexpression in *Mecp2*-deficient male has enhanced survival and locomotor utility in these mice [171].

Exogenous BDNF is not a practical therapeutic choice for a neurodevelopmental disorder like Rett Syndrome as it does not cross the blood–brain barrier (BBB) [184]. However, Insulin-like Growth Factor 1 (IGF-1) is a major activator of the same signaling pathways as BDNF [185,186] with the ability to cross the BBB [187] and therefore has been used in different clinical trials as a therapeutic agent for RTT [188,189,190,191,192]. These trials have shown variations in results [193], which could be due to the complexity of RTT condition.

#### 7.1.5. BDNF and Cellular Origin of Detection in the Human Brain

Although BDNF was first isolated from the brain [194], it does not necessarily prove that neurons are its primary cellular origin. Indeed, it has been almost 20 years that in vitro investigations have revealed that endothelial cells of cerebral microvasculature would also produce BDNF [195,196]. After being overlooked for years, more recent in vivo research experiments have shown that BDNF is produced by endothelial cells of the adult cerebrovasculature [197,198]. Even a 50-time greater BDNF expression level has been reported from cerebral endothelial cells compared to cortical neurons [199]. Based on a few other research studies, removal of cerebral endothelial cells significantly decreases BDNF levels in the brain [200]. Furthermore, different studies have shown that glia including astrocytes and oligodendrocytes synthetize and release BDNF [201,202]. The noticeable astroglial/endothelial pattern of BDNF staining that we recently reported [180] spreads the previous findings in the animal models and cell culture systems to different regions of RTT and control human brains. At the same time, it adds more questions to the role of MeCP2-BDNF cross-talks in RTT pathophysiology because most model systems have focused on neurons as the main source of BDNF, which has been complemented by our recent findings [110].

Besides the cellular source of BDNF, its impairment in RTT patients is another controversy that a recent study from our team has approached from a postmortem human brain angle. In our recent study, we showed lower *BDNF* transcripts in RTT brains. However, BDNF protein (investigated by Western blot, ELISA, and IHC) did not follow the same trend. Surprisingly, we detected higher BDNF levels in the Purkinje cells of an RTT cerebellum. In this regard, we found a low predictive value for the transcripts relative to the matching protein [110,180], which is not uncommon and could result from a complex regulatory mechanism in the human brain [203]. However, it is noticeable that the anti-BDNF antibody that we used for Western blot and IHC was capable of detecting pro-BDNF and the mature protein. The coating antibody of the ELISA kit also detected both mature BDNF and pro-BDNF. While the Western blot experiment differentiates between pro-BDNF and mature BDNF by molecular size, ELISA and IHC show the combination of both protein forms.

Considering the inhibitory character of Purkinje cells as GABAergic neurons, we might relate more intense immunolabeling of these cells in RTT brains to a higher level of pro-BDNF, which has a reverse function compared to the mature BDNF [148]. From the two approach of studying BDNF in the human brain by quantitative Western blot and qualitative IHC analysis, we reported that lower levels of mature BDNF protein and higher levels of pro-BDNF in the cerebellum are actually in line with the IHC findings [110,180]. BDNF detection rises upon hypoxia or conditions like neuroinflammation [204,205]. Such conditions contribute to the pathogenesis of different neurological disorders but have not yet been explored in postmortem human RTT brain tissues. Our recent report not only contributed to the pathobiology of RTT in clinically relevant patient brain tissues but also is important from a therapeutic point of view, mainly by considering the endothelial cells as a major source of BDNF in human brains that might allow us to circumvent the blood–brain barrier and to focus on increasing BDNF levels in the brain through its nonneuronal sources.

### 7.2. MicroRNAs

MicroRNAs (miRNAs) are members of the noncoding RNA family with a length of approximately 22–23 nucleotides that can regulate a vast number of biological events through gene silencing. In the genome, miRNA genes can be localized within the introns of coding genes (host genes) or in areas without known coding activity either as a single gene or as clusters of genes [206]. The process of miRNA biogenesis is tightly controlled in a temporal and spatial manner, and its deregulation has been shown to be associated with several human diseases [207].

#### 7.2.1. Role of miRNAs in Central Nervous System Development

There are still many points to be clarified about how miRNAs regulate their target genes. However, we know that miRNAs affect the process of nervous system development during embryonic patterning and into neural differentiation and plasticity [208]. More recent studies have also shown the important role of miRNAs in adult synaptic plasticity and cognition [206]. Time-specific spatially restricted or cell type-specific miRNAs can play roles in cell fate determination of neuronal precursor cells toward neurons or glial cells. This happens mainly during embryonic development for neurons and continues early postnatally for glial differentiation. Regulatory RNAs such as miRNAs are also associated with adult neurogenesis in specific adult brain regions such as subgranular zone with roles in learning and memory. In addition, miRNAs are involved in glial and neural cell type determination. These regulatory molecules also play a role in the migration of newly formed neurons to their specific destinies and neuronal polarization, which refers to functional separation of neuron processes to axonal and dendritic compartments. Axonal formation and dendrite branching are other areas that neuronal miRNAs may regulate. They also affect maturation of neurons through connection with proper targets. The dynamic structure and function of synapses give them the ability to respond to external stimuli. This process recognized as synaptic plasticity is also affected by miRNA reaction to activity-dependent pre- and postsynaptic physiology [208].

In summary, several studies have highlighted the regulatory role of miRNAs in every aspect of neural development, and their impairments have been observed in several neurologic disorders such as schizophrenia, autism, and RTT [206].

#### 7.2.2. The Role of *miR132* and its Effects on Neural Structure and Function

Among the many miRNAs that are present in the central nervous system, *miR132* is expressed in an activity-dependent manner. This microRNA has an extremely conserved sequence among vertebrates and is regulated by CREB as the transcription factor [209]. The *miR132* not only affects neuronal morphology, but it also controls neuronal function. One supporting evidence for such an activity comes from a study that shows that BDNF-induced axonal branching in mouse retina can be promoted by *miR132* [210]. This activity-regulated miRNA also regulates dendritogenesis in mice and chicks [211,212]. The *miR132* also plays a role in dendritic spine morphogenesis, affecting synaptic plasticity [213,214]. Deregulation of *miR132* has been shown to be associated with different neurological disorders, as expected from its role in neuronal development and function. Downregulation of *miR132* in the brain of patients with Huntington’s disease, schizophrenia, and bipolar disorder are a few examples [215,216].

The location and function of *miR132* is not limited to neurons. Its level changes in immune-related contexts and there is increasing evidence for *miR132* involvement in inflammatory processes. For example, inflammatory conditions induce the *miR132* level in different cell types such as monocytes, mast cells, and lymphatic endothelial cells. There are also reports suggesting that hormone and nutrition condition can regulate *miR132* [217]. Furthermore, the function of *miR132* encompasses areas like tumorigenesis. For instance, *miR132* level has been shown to decrease in pancreatic cancer or to increase in chronic lymphoblastic leukemia when compared to the noncancerous condition [218,219].

#### 7.2.3. Homeostatic Regulation of MeCP2 by *miR132*

Multiple polyadenylation sites of the *MECP2*/*Mecp2* gene result in transcription with short (approximately 1.8 kb) or long (approximately 10 kb) 3′UTRs. The main transcript in the brain is the longer form with highly conserved miRNA response elements (MRE) for several miRNAs including *miR132*. The smaller transcript does not have these sites [220]. While the basal level of *miR132* is low before birth, it has been shown that this microRNA contributes to BDNF-mediated neurite outgrowth in rat neonatal neurons. On the other hand, *miR132* introduction into rat primary cortical neurons negatively affects the protein level of MeCP2 [221]. Forskolin and KCl treatment both induce *miR132* through the CREB pathway, leading to a decreased MeCP2 level. The unchanged level of mRNA in this study is in favor of a post-transcriptional effect [169]. The authors also showed that MeCP2 overexpression as well as *miR132* blocking could increase *BDNF III* transcript while *BDNF I* without binding site for MeCP2 or *miR132* MRE did not change. In addition, in the Jaenisch *Mecp2*-knockout mice, both *Bdnf IV* and *miR132* were decreased. Findings from the same authors that show MeCP2 increases *BDNF* levels together with reports that BDNF activates *miR132* expression leads to the suggested homeostasis network that MeCP2 induces BDNF, which itself induces *miR132* that represses MeCP2 protein. From several studied miRNAs that can bind the 3′UTR of *Mecp2*, *miR132* is the only one which is enriched in the brain [169].

Another study has shown that, during the fetal stages, miRNAs other than *miR132* (for example, *miR483*) can suppress MeCP2 protein. However, *miR132* can fine-tune the MeCP2 level in the postnatal stages [222]. MeCP2 regulation by *miR132* through binding to evolutionary conserved binding sites has been studied in animal models in different contexts from RTT [169] to drug abuse [223] and pain transmission [224]. However, the limited number of studies on human cells or brain samples is not consistent with the results from animal models [222]. The two main isoforms of MeCP2 are identical in 96% of their amino acids. The MeCP2E1 isoform is slightly longer (498 amino acids in human) with 21 unique N-terminal amino acids. The MeCP2E2 isoform, which is 12 amino acids shorter, has 9 unique N-terminal amino acids [46,107]. While the MeCP2E1 isoform is present in all vertebrates, the MeCP2E2 isoform is only found in mammals [37]. The predominance of the MeCP2E1 isoform in the brain and the brain region-specific expression of the two isoforms has already been shown in mice brain by independent groups including us [40,107,109]. In addition, the half-lives of the two isoforms have been predicted to be very different [225].

The fact that two isoforms of MeCP2 are highly similar and observations where the capacity of MeCP2E2 overexpression prevents key RTT-like phenotypes in RTT mice models [49] point towards a functional overlap between the two isoforms. However, the facts that mutations that only affect *MECP2E1* can cause RTT and that *Mecp2e1*-specific knockout can generate RTT mouse models [226,227] suggest that MeCP2E2 is not capable of compensating for the lack of MeCP2E1 in vivo.

Due to limited availability of the human brain samples and technical difficulties in controlled assessments of these samples, the distribution and level of the two MeCP2 isoforms have remained largely unexplored in the human brain. Moreover, research into MeCP2 regulatory systems has mainly targeted animal models. A regulatory loop composed of MeCP2, BDNF, and *miR132* has been suggested to exist in the rat brain. The *miR132* is a neuronal-specific microRNA (highly conserved among vertebrates [217]) that inhibits MeCP2. Expression of this microRNA is induced by BDNF, which is controlled itself by MeCP2 [169]. Although a well-received study in the field, the conservation of this regulatory loop has not been studied in the human brain.

## 8. Lessons Learned from the Human Brain on MeCP2-BDNF-*miR132* homeostasis Regulatory Components

The earliest MeCP2 expression in the normal human brain is at 10 gestational weeks, reported in the brain stem and cerebral cortex in the subcortical and Cajal–Retzius neurons. MeCP2 will appear subsequently in the thalamus, in the midbrain, and in the basal ganglia. The MeCP2 levels in the hippocampus and cerebellum are not high and show lower levels during early development. However, upon cellular maturation in neurons, most of these cell types in these regions express MeCP2. That might explain the delay in clinical manifestation of RTT in the course of development [40]. The MeCP2 protein shows a nuclear pattern of distribution, while slight cytoplasmic detection has been reported in some neuronal cells. Post-translationally modified proteins have been suggested to be the source of this cytoplasmic fraction. Since the earliest immunohistochemistry (IHC) studies, a consistent challenge has been the inconstant level of MeCP2 staining within the same neuronal cell types [18]. Laser scanning cytometry has confirmed that there are cells with both low and high MeCP2 levels [228]. It is possible that such detected differences result from either neuronal activity or the possibility of postmortem degradation of the MeCP2 protein [18,40]. Regarding other protein labeling, some increase in the glial fibrillary acidic protein (GFAP) has been reported in RTT brains. However, it is not clear whether this is a primary or secondary phenomenon to the main RTT pathology [18].

Recently, our team reported that, in the human RTT brain, *MECP2* isoforms are significantly reduced compared to age-and sex-matched controls [110]. Our findings were in the same line with earlier research reporting significantly lower levels of *MECP2* transcripts [180], impaired structure and function of MeCP2 protein in neuronal [18,58,229], or nonneuronal RTT samples (e.g., peripheral blood) [230]. However, we did not find a clear association between the *MECP2E1/E2* transcript and MeCP2E1/E2 protein levels [110]. BDNF and its precursor did not show any concordance with the *BDNF* transcript either. In general, the multi-layer regulation of protein levels in the human brain can partly explain the low projecting value of transcript correlation for the corresponding protein(s) [203]. Post-transcriptional and post-translational control mechanisms, in addition to cell type-and tissue-specific monitoring systems that control protein stability, turn-over, and expression, could be part of the possible causes [40].

Our recent analysis of human postmortem RTT brains showed that *BDNF* has similarly significant lower transcript levels compared to controls, but surprisingly, the protein level was comparable to control brains. However, our report on the formalin-fixed brain samples revealed yet another layer of complexity about the MeCP2-BDNF regulatory network. Based on our recently reported results on BDNF labeling, different cells of the brain were positive for BDNF. Such cells included astrocytes, neurons, and endothelial cells. All these cells can possibly contribute to MeCP2 homeostasis as sources of production and expression of BDNF. Hence, it is probably too naïve to think that one simple regulatory mechanism controls MeCP2-BDNF homeostasis without taking into account the cell type of origin for each protein.

In contrast to the negative regulatory role of *miR132*, supported by experiments in rodents [169], we observed that lower *MECP2E1/MECP2E2* transcripts in the human RTT brains accompany a lower level of *miR132* in the amygdala, hippocampus, and frontal cortex but not in the cerebellum. Our findings in general did not suggest the existence of a conserved role for *miR132* on MECP2/MeCP2 homeostasis in the human brain. However, our data pointed out that cerebellum is different from the other brain regions by showing similar levels of *miR132* in RTT and control brains [110]. The differences that we observed inter-regionally in the MeCP2-BDNF-*miR132* homeostasis regulatory network in human brains could have partly originated from different cellular compositions of each brain region versus the other part of the brain, suggesting that there are more complex regulatory mechanisms in each brain region with their specific functional role in the context of a whole human brain without a unifying mechanism to control this regulatory network.

We also showed that postmortem delay could have affected the results of previous human RTT brain studies regarding MeCP2 immunostaining of neurons [180]. Our research also provided more evidence for changes in astroglial cells in the course of Rett Syndrome [180].

## 9. Therapeutic Strategies for RTT

The monogenic character of classical RTT and the reversibility of the symptoms in preclinical models have brought optimism to the therapeutic research for this devastating disease. The availability of rodent models showing quantifiable symptoms such as abnormal breathing makes evaluations of therapies more translatable when compared to the behavioral disorders of more common conditions such as non-symptomatic autism [231]. Our understanding of MeCP2 function and the outcome of its deficiency on the function of neuronal circuit and behavior is the basis for potential therapeutic approaches. Such strategies would include (1) molecular methodologies to replace gene/protein or to reactivate the wild-type allele from the epigenetically silenced and inactive X-chromosome and (2) pharmacologic strategies tailored towards MeCP2 downstream molecules and events (target genes or molecular functions) to restore their role in neuronal circuits [232].

### 9.1. Molecular Treatments and Gene Dosage Concerns

**Figure 3:**
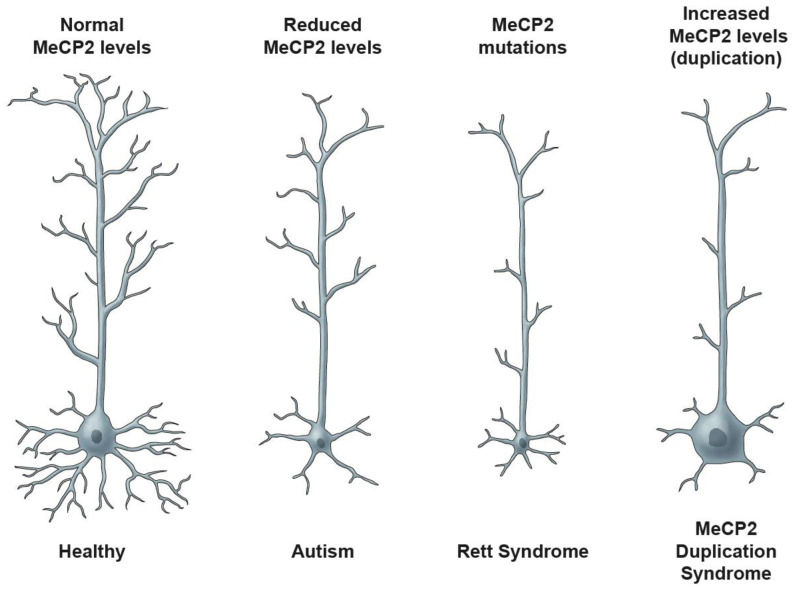
MeCP2 levels determine the phenotypic characteristics of neurons. A simplified representation of neuronal morphology with respect to MeCP2 level, soma size, neurite formation, and association with human disease is shown.

An excess of MeCP2, a condition similar to what happens in boys with *MECP2* Duplication Syndrome (MDS), leads to RTT-like neurological dysfunction presenting with seizure and hypoactivity [233,234]. Therefore, any molecular therapy that targets *MECP2* directly to normalize the protein must keep the level of protein within its narrow acceptable limits by avoiding MeCP2 over dosage [231]. In general, neurons with proper MeCP2 levels would have normal neuronal structures while neurons with reduced MeCP2 levels are associated with autism. On the other hand, neurons with MeCP2 loss- or gain-of-function are affected in RTT and MDS, respectively (Figure 3). In fact, there might be a relation between the MeCP2 level of expression or genetic mutation with neuronal structure characteristics that has been evidenced by studies on mouse models of RTT Syndrome and detected deficiencies in synaptic plasticity using in vitro and in vivo murine and human systems [191,235,236,237,238,239,240,241,242,243,244]. Figure 3 provides a simple illustration of the neuronal structures in MeCP2-associated neurodevelopmental disorders based on the reported impact of MeCP2 levels in the indicated in vitro and in vivo systems [191,235,236,237,238,239,240,241,242,243,244].

### 9.2. Activating MECP2 on the Inactive X-Chromosome

This novel method has been already studied in another neurodevelopmental disorder (Angelman syndrome), and the *Mecp2*-*EGFP* fluorescent reporter mouse is a useful tool for high-throughput, small-molecule screening. Other than the cost and stability concerns for this procedure, the availability of active and safe compounds that diffuse across the blood–brain barrier is another limitation in restoring MeCP2 levels across the relevant cell types. Furthermore, targeting *MECP2* specifically or the entire inactive X is another concern in this method [231,245,246].

### 9.3. Gene-Editing Strategies

The first attempt to study a gene therapy for Rett Syndrome was reported over a decade ago by Dr. Rastegar and colleagues [111]. We showed that viral vectors carrying the endogenous *Mecp2* promoter could be effective for gene therapy delivery by recapitulating the endogenous expression pattern of MeCP2 in neurons and glia [111]. Editing or replacement an abnormal gene has also become available through adeno-associated virus (AAV) vectors. In this case, IV delivery of an AAV9 vector carrying the *Mecp2* cDNA has to some extent normalized the symptoms of male and female RTT mice [231,247]. Delivering *Mecp2* expression homogenously and within the narrow normal range is one of the main challenges for gene therapy [231]. About 35% of RTT patients with in-frame premature stop codons might benefit from compounds that allow the readthrough of nonsense mutations [248]. It has been shown to be effective in cultured R168X (most common RTT-causing truncated mutation) mouse fibroblasts [109,249].

### 9.4. Challenges of Protein Replacement

Homogenous and continuous delivery of appropriate level of MeCP2 across the BBB is the major challenge for protein replacement. Ensuring adequate penetration of the protein at the cellular and subcellular levels up to the nucleus and making sure that posttranslational modifications occur in a regular manner are other obstacles to overcome in this method [231].

### 9.5. Targeting Downstream Signaling Pathways of MeCP2

Classical neurotransmitter and neuromodulator signaling; growth factor signaling such as BDNF and IGF-1; as well as metabolic signaling, cholesterol biosynthesis and mitochondrial function are the main known MeCP2-targeted pathways [250,251]. A drug prepared for one pathway might not treat the full spectrum of RTT symptoms. However, ameliorating one main symptom such as breathing abnormalities may have a considerable impact on the quality of life for RTT patients [231].

### 9.6. Clinical Trials

There are several challenges in a clinical trial for a rare disease like RTT. A vast number of RTT-associated mutations and a limited pool of participants are two of the obstacles. From several ongoing or completed trials, only rare ones are parallel, randomized, double blind, and placebo-controlled, and none has reached the level to be used in practice [6,231].

## 10. Closing Remarks

Today, after almost three decades since the discovery of MeCP2, with its relation to Rett Syndrome known for over two decades, we are still searching for an effective therapeutic strategy for this devastating disorder. In this regard, intensive efforts from basic scientists and clinician scientists have advanced our understanding very significantly. Animal and cellular models have helped us to take steps towards understanding the pathophysiology of the disease and mechanistic approaches for therapeutic interventions. However, needless to say, research on postmortem human brain tissues, despite its difficulties and limitations, can provide us with invaluable information about the real complex disease condition. Clearly, due to the many regulatory roles of MeCP2 in the brain, the more we proceed, the more we face new questions and challenges for finding an ultimate cure for MeCP2-associated neurodevelopmental disorders such as Rett Syndrome.
